# Hippocalcin-Like 1 blunts liver lipid metabolism to suppress tumorigenesis via directly targeting RUVBL1-mTOR signaling

**DOI:** 10.7150/thno.75936

**Published:** 2022-10-24

**Authors:** Tao Chen, Zhiqing Yuan, Zhou Lei, Jinlin Duan, Junyan Xue, Ting Lu, Guouan Yan, Lei Zhang, Yanfeng Liu, Qiwei Li, Yonglong Zhang

**Affiliations:** 1 United International Laboratory, Department of Clinical Laboratory, Sixth People's Hospital, School of Medicine, Shanghai Jiao Tong University, Shanghai, China.; 2 Department of Biliary-Pancreatic Surgery, Renji Hospital, School of Medicine, Shanghai Jiao Tong University, Shanghai, China.; 3 Department of Hepatobiliary Surgery, First Affiliated Hospital of Bengbu Medical College, Bengbu, 233000, Anhui Province, China.; 4 Department of Pathology Affiliated Tongren Hospital, School of Medicine, Shanghai Jiaotong University, Shanghai, China.; 5 Institutes of Biomedical Sciences of Shanghai Medical School, Fudan University, Shanghai, China.; 6 Renji-Med-X Clinical Stem Cell Research Center, Ren Ji Hospital, School of Medicine, Shanghai Jiao Tong University, Shanghai, China.

**Keywords:** Hippocalcin-Like 1, RUVBL1, mTOR addiction, fatty acid biosynthesis, Cholesterol synthesis

## Abstract

**Rationale:** Hepatocellular carcinoma (HCC) is one of the most severe cancers worldwide, with few effective targeted therapies for HCC. Lipid metabolic reprogramming is emerged as a hallmark of cancer metabolism that guides response to antitumoral therapies. Such lipid metabolic alteration in cancers is critically regulated by the mammalian target of rapamycin mTOR, which is considered as a promising therapeutic target. Despite efforts, mTOR inhibitors (mTORi) have produced limited response clinically, partly due to incomplete knowledge of mTORC1 addiction in cancers.

**Methods:** CRISPR-Cas9 system was used to establish *Hpcal1* null mice. The liver cancer model in mice was generated using *Hpcal1*-deficient mice with diethylnitrosamine (DEN) /CCL4 or MYC/Trp53^-/-^ via hydrodynamic tail-vein injection. RNA-sequencing (RNA-seq) was used to identify potential signaling pathways. The expression of HPCAL1 and mTOR signaling were determined using quantitative polymerase chain reaction (qPCR), western blot and immunohistochemistry. The role of Hpcal1 in liver tumorigenesis and its response to mTORi was assessed by CCK-8 measurements, colony formation assay and in mouse model.

**Results:** In this study, we identified hippocalcin-like protein 1 (HPCAL1) as an important negative regulator of *de novo* lipid biosynthesis and mTOR signaling activation, limiting liver tumorigenesis and establishing a metabolic vulnerability of HCC in mice. Genetic loss of HPCAL1 rendered HCC mTORC1-addicted and sensitive to mTORi AZD-8055 *in vitro* and *in vivo*. Importantly, HPCAL1 expression was inversely correlated with the levels of mTOR phosphorylation and several critical lipid biosynthesis enzymes in human specimens. Mechanistically, HPCAL1 directly bound to RuvB Like AAA ATPase 1 (RUVBL1), inhibiting the assembly of TEL2-TTI1-TTI2 (TTT)-RUVBL complex and subsequent leading the mTOR signaling suppression.

**Conclusion:** We uncover a metabolic vulnerability and mTOR addiction in HCC with HPCAL1 loss that provides a selective therapeutic window for HCC with mTORC1 hyperactivation using mTORi.

## Introduction

Hepatocellular carcinoma (HCC) is the third most frequent cause of cancer-related death worldwide and is associated with a significant clinical, economic, and psychological burden 1-3. Although emergence of several new targeted therapies for the treatment of HCC has shown promise and therapeutic benefit over past 5 years, the long-term patient survival times remains to be improved 1, 4, 5. Developing additional novel therapeutic drugs or understanding the underlying mechanism of tumor progression and therapeutic resistance to available targeted drugs will be helpful to identify a selective therapeutic window and improve the survival of patients with HCC 6.

Lipid metabolic reprogramming is an established hallmark of cancer metabolism that provides lipids and lipid precursors for membrane synthesis, signaling molecules and energetic substrates for rapidly growing tumor cells that are continuously adapting to unfavorable conditions, including therapeutic stresses 7-9. Emerging evidences have suggested that aberrant lipid metabolism imposes a potential metabolic vulnerability that could be exploited to guide response and resistance to antitumoral therapies 8.

The mammalian target of rapamycin (mTOR) is a master regulator of lipid biosynthesis and lipolysis via its diverse downstream signaling pathways 10-12. Constitutive mTORC1 hyperactivation is frequently detected in cancers that establish mTOR addiction to drive metabolic reprogramming and sustain rapid growth and survival, and has been considered an attractive therapeutic target 13, 14. This addiction to mTORC1 signaling in certain cancers has created a substantially increased sensitivity to mTOR inhibitors (mTORi), which inspired the clinical development of mTORi 15. Indeed, targeting mTOR signaling for mTOR-addicted tumors has shown beneficial effects in preclinical models 16-18. However, only modest or short-term clinical response of several tumors to mTORi was observed in some clinical trials 19-21. This is partly due to the incomplete understanding of the mechanisms underlying mTOR activation and mTORC1 addiction in cancer cells, which compromises the identification of effective biomarkers for predicting potentially responsive patients and guiding clinical implications.

mTOR is found in two complexes (mTORC1 and mTORC2), each with distinct protein components as well as substrates 22, 23. Activation of mTORC1 occurs at the lysosomal membrane by Rag GTPase heterodimers and is known to be mediated by the small GTPase Ras homologue enriched in brain (RHEB), which is itself inhibited by the tuberous sclerosis heterodimer (TSC1/2) in response to amino acids and glucose 12. In addition, recent studies revealed that mTORC1 assembly, lysosomal localization and activation are regulated by the TEL2-TTI1-TTI2 (TTT)-RuvB Like AAA ATPase complex, which is independent of TSC1/2 and Rag upon energy stress 24-28. Further study demonstrated that RUVBL1/2 proteins are pivotal for the vulnerabilities of mTORC1-addicted cancer cells. Targeting RUVBL1/2 causes synthetic lethality in mTORC1-hyperactive cancer cells, providing a selective therapeutic opportunity for tumors with high mTORC1 activation 27.

HPCAL1 is a neuronal calcium sensor with three EF-hand domains that belongs to a member of the visinin-like proteins (VILIP) superfamily, which include visinin-like protein 1 (VILIP-1) (VSNL1), VILIP-2 (HPCAL4), VILIP-3 (HPCAL1), hippocalcin (HPCA), and neurocalcin δ (NCALD). VILIP-1 is identified a tumor suppressor that inhibits the epidermal-mesenchymal transition in esophageal squamous cell carcinoma and non-small cell lung carcinoma 29, 30. Other members in this family have rarely been reported in cancers. We have previously identified that HPCAL1 was frequently lost in HCC, resulting cell cycle progression and hepatocarcinogenesis via modulating Cyclin-dependent kinase inhibitor 1 p21^WAF1/CIP1^ stabilization 31. By contrast, HPCAL1 was reported to facilitate the differentiation and proliferation of glioblastoma via distinct mechanisms 32, 33. In this study, we have uncovered that HPCAL1 serves as an important negative regulator of* de novo* lipid and cholesterol biosynthesis and tumor progression in mice. Through binding to RUVBL1/2, HPCAL1 diminishes the assembly of TTT-RUVBL complex and mTOR signaling activation and renders HCC therapeutic resistant to mTORi.

## Materials and Methods

### Sample collection

90 cases of pathologically examined liver cancerous tissues were collected from Renji Hospital between 2014 and 2018. Tissue microarrays (TMA) containing these cases were prepared by Shanghai ZUOCHENG Ltd. All experiments and analyses were conducted with the understanding and written consent of each participant. All manipulations were performed under the approval of the Research Ethics Committee of Renji Hospital, School of Medicine, Shanghai Jiao Tong University.

### Generation of Hpcal1^-/-^ mice

C57BL/6J mice were constructed by CRISPR-Cas9 system. Briefly, four sgRNAs were designed to target the Hpcal1 gene. The oligonucleotide sequences are as follows: sgRNA1, 5′- AAGGGTCATTCAATCCCCACAGG -3′; sgRNA2, 5′- GGGTCATTCAATCCCCACAGGGG -3′; sgRNA3, 5′- TGCAATCTGAACCCTTCCAATGG -3′; sgRNAs were then cloned into T7-cas9 vector and linearized with for *in vitro* transcription using the mMACHINE T7 Ultra kit (Ambion), followed by purification using RNeasy Mini kit (Qiagen). A mixture of Cas9 mRNA and four sgRNAs was injected into the cytoplasm and male pronucleus of the zygote, which were transferred into pseudopregnant C57BL/6J female mice to yields F0 generation mice. Genomic DNA was isolated from tails and analyzed by PCR amplification using the following primers: 5′-TGTCCCCTTATACCACGATTT-3′ and 5′-CCAACCCTCACTGCTTTTG-3′. All mice were maintained in specific pathogen-free facilities and housed in single-sex cages at 20 ± 2 °C with 40-60% humidity and a 12-h light/12-h dark photoperiod. All animal experiments were performed according to the National Institute of Health Guide for the Care and Use of Laboratory Animals under the approval of the Research Ethics Committee of Renji Hospital, School of Medicine, Shanghai Jiao Tong University.

### Diethylnitrosamine (DEN) /CCL4 liver cancer model

For the HCC model induced by DEN and CCl4, 2-week-old male mice were intraperitoneally injected with 25 mg/kg DEN. At age of 8 weeks, mice were injected intraperitoneally with 25% CCl4 (2 ml/kg) in corn oil once a week for additional 12 weeks. Mice were then killed, and livers and sera were collected for subsequent analysis.

### MYC/Trp53^-/-^ liver cancer model via hydrodynamic tail-vein injection

8-week-old was injected with 2 mL sterile 0.9% NaCl solution/plasmid mix containing 12 μg of MYC-Luc, 12 μg of sg*p53*, 12 μg of sgCON or sg*Hpcal1* or sg*Ruvbl1* and 3 μg of SB13 transposase-encoding plasmid via tail vein within 5 to 7 s 34. 30 days post injection, mice were then killed, and livers were collected for subsequent analysis. For survival analysis, mice were observed regularly every day after injection for 30 days until all mice were dead. Survival curves were constructed according to the survival times. For sg*Hpcal1*, four tandem sgRNA (same as above) driven by separate U6 promoters were prepared in plenti-CRISPR V2 (Addgene Plasmid 49535). For sg*Ruvbl1*, three tandem sgRNA by separate U6 promoters was used. The sgRNA sequence was as follow: sg*Ruvbl1*-1, 5'-CACATCTAGATAAACTTCGA-3'; sg*Ruvbl1*-2, 5'-GCCGCGCATTAGCCACATCC-3'; sg*Ruvbl1*-3, 5'-GCTCGGATCTTAATGATCTT-3'.

### Analysis of liver enzymes

Serum analyses of ALT and AST from WT and *Hpcal1* mice were determined using an Alanine Aminotransferase Assay kit and Aspartate Aminotransferase Assay kit (Nanjing Jiancheng Ltd., China).

### Hematoxylin and eosin (HE), immunohistochemistry (IHC) and Oil red O staining

Formalin-fixed and paraffin-embedded mouse livers or human liver cancers were analyzed by HE as previously reported 35, 36. For IHC staining, liver sections were preincubated in normal goat serum for 15 min, and were incubated with anti-phos-mTOR (Cell signaling, #2976,1: 50), anti-phos-4EBP1 (Cell signaling, #2855, 1:100), anti-HPCAL1 (Sigma, SAB1307075; 1:200), anti-SCD1 (Abclonal, A16429; 1:400), anti-ACSS2 (Proteintech, 16087-1-AP, 1:400), RUVBL1 (Proteintech,10210-2-AP,1:400) and Ki-67 (Proteintech, 27309-1-AP, 1:5000) at room temperature. Two hours later, secondary antibody (JACKSON, 111-035-003, 1:500) was used, followed by incubation with DAB Chromogen dilution solution. For Oil red O staining, Oil Red O (0.5% (v/v) in isopropanol) was diluted with water (3:2), filtered through a 0.45 mm filter, wished 3 times with PBS and fixed for 30 min with 4% (v/v) paraformaldehyde (PFA), then the fixed cells were incubated with filtered Oil Red O for 1 h at RT. Quantification of Oil red O staining was performed by Image J software as previously described 37.

### IHC staining and scoring

IHC staining was independently assessed by two experienced pathologists. The staining intensity was graded from 0 to 2 (0, no staining; 1, weak; 2, strong). The staining extent was graded from 0 to 4 based on the percentage of immunoreactive tumor cells (0%, 1%-5%, 6%-25%, 26%-75%, 76%-100%). A score ranging from 0 to 8 was calculated by multiplying the staining extent score with the staining intensity score, resulting in a low (0-4) level or a high (6-8) level for each sample.

### Reagents and Antibodies

AZD-8055 was purchased from MedChemExpress (Shanghai, China). Antibodies for western blotting against HPCAL1, ACSS2, RUVBL1, GAPDH, his tag and HA tag were obtained from Proteintech (Wuhan, China). Antibody against phos-mTOR, and phos-4EBP1 and phos-4EBP1 were purchased from Cell Signaling Technology (USA). SCD1 antibody was obtained from ABclonal (Wuhan, China). HPCAL1 antibody for IHC staining and anti-FLAG M2 gel was purchased form Sigma-Aldrich (USA).

### Cell culture, RNA interference, Cell proliferation and Colony Formation Assay

Human Embryonic Kidney (HEK) 293T and Huh7 were purchased from Cell Bank of Shanghai Institutes of Biological Sciences. HEK-293T and Huh7 were maintained in DMEM high glucose medium (Shanghai BasalMedia Technologies) in 5% CO2 at 37 °C incubator. All cell lines underwent detection for mycoplasma contaminants using Mycoplasma Detection Kit (40611ES25, YEASEN, Shanghai, China) and confirmed mycoplasma-negative. STR authentication were performed to confirm the identity of Huh7. RUVBL1, RUVBL2, RPTOR, RICTOR and HPCAL1 knockdown was achieved by RNA interference using short interfering RNA (siRNA) and lentiviral vector-based shRNA, respectively. The siRNA sequence used was as follows: siRUVBL1, 5'-AAGGAACCAAACAGTTGAAACTG-3'; siRUVBL2, 5'-TAACAAGGATTGAGCGAAT-3'; siRPTOR: 5'-GGACAACGGCCACAAGTACTT-3'; siRICTOR 5'- AGACAAGGCCAATCTTCATGC-3'; shHP#1, 5'-GGTGACATGCAGGGTTCAAGT-3' and shHP#2, 5'-GCCGCTTGCACGTATAGATAC-3'. shRNA lentivirus particles were prepared as previously described 38. Cell proliferation was performed by CCK8 assay in Huh7 cells with indicated doses of AZD-8055, and all the experiments were performed in triplicate with each group in quintuplicate as previously reported 38. For colony formation, cells infected with indicated lentivirus was prepared in suspension medium with cell density of 1 × 10^3^ cells/mL. 1-3 mL cell suspension medium were seeded into 6-well plates in triplicate overnight. Cells were then treated with for indicated doses of AZD-8055 for additional 2-3 weeks. Plates were washed gently twice with PBS, and fixed with 4% methanol for 30 min. The number of colonies was counted and statistically analyzed.

### Purification of HPCAL1 Interacting Proteins and Protein Identification

Huh7 cells infected with vector or FLAG-HPCAL1 were lysed to a homogenous mixture. The supernatant was incubated with 20 μL FLAG-M2 gel overnight on a gentle rotating machine. The resulting pellet was washed five times with RIPA buffer (50 mM Tris (pH 7.4), 250 mM NaCl, 1% TritonX-100, 1% sodium deoxycholate, 1 mM EDTA and 20% glycerol), and was then eluted with 500μL FLAG peptide (5 mg/mL) in TBS (10 mM Tris HCL, 150 mM NaCl, pH7.4). The eluates were further recovered using ultrafiltration spin columns (3 kDa), and were subjected to sodium dodecyl sulfate-polyacrylamide gel electrophoresis and Coomassie blue staining. Protein band cutting, in-gel trypsin digestion, peptide extraction, LC-MS/MS analysis, and protein identification were performed as previously described 31, 38.

### Statistical analysis

All statistical analyses were carried out using GraphPad Prism 7 or SPSS for Windows 25 software (SPSS Inc., Chicago, IL, USA). The comparisons of measurement data between two groups were performed using unpaired t test. The comparisons among three or more groups were firstly performed by One-Way ANOVA test if the variation between groups were comparable. Overall survival was evaluated by the Kaplan-Meier survival curve and the Log-rank test using GraphPad Prism 7. The correlation of the two proteins were examined by Pearson's correlation test. T statistical tests and p-values were two-sided. Differences were considered significant with a value of P < 0.05.

## Results

### *Hpcal1* deletion promotes Hepatocarcinogenesis

**Figure 1:**
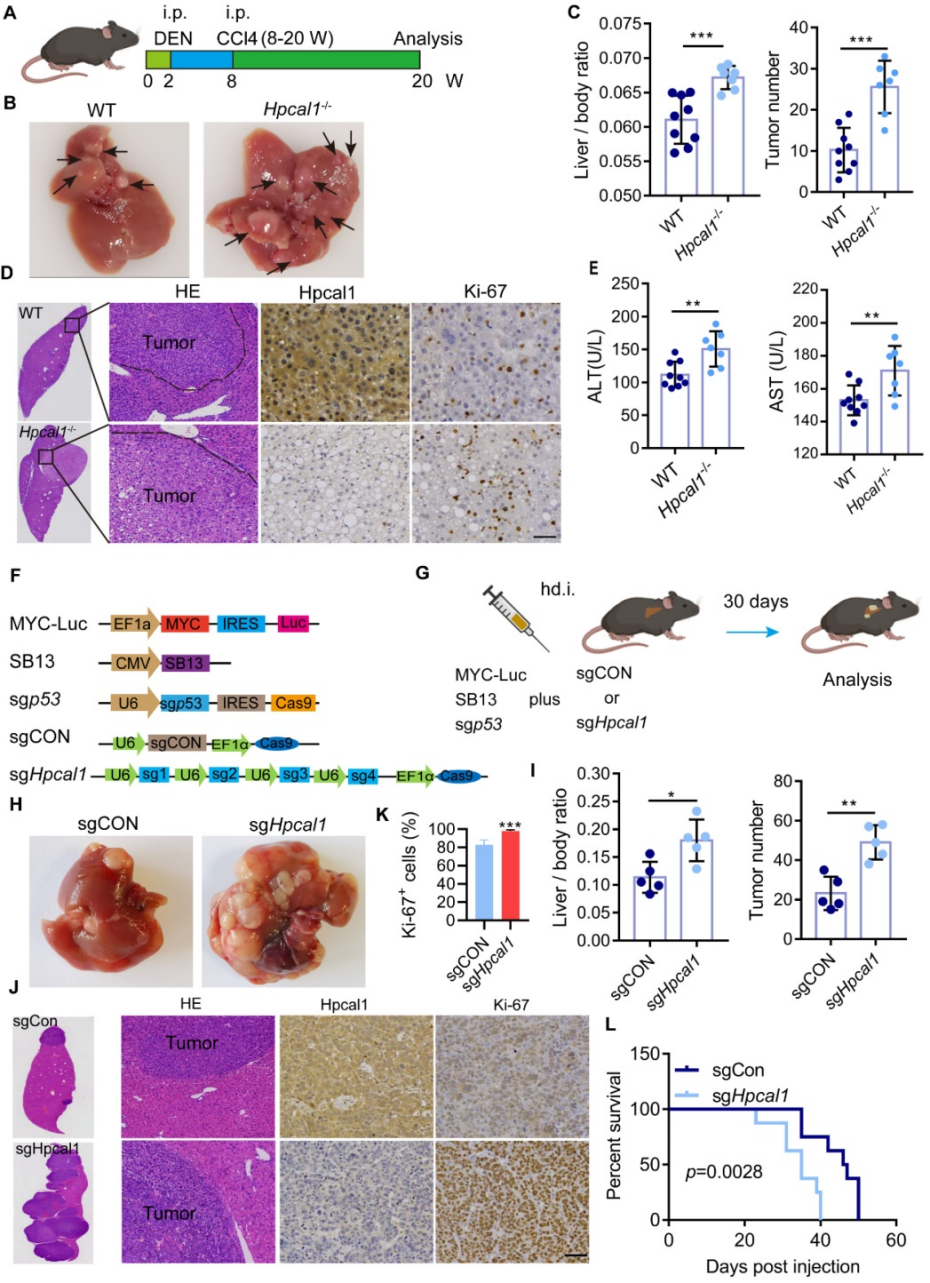
** Genetic loss of Hpcal1 results in enhanced liver tumorigenesis. (A)** Scheme of DEN/CCL4 induced murine liver cancer model. Two-week old mice were intraperitoneally (i.p.) injected with DEN at dose of 25 mg/kg in saline. At age of eight weeks, mice were administrated with CCl4 in a volume of 2 ml/kg body weight (CCL4 was mixed with corn oil at ration 1:3 once a week for additional 12 weeks. W, week. This figure was drawn by using BioRender (JX24G8PWZC). **(B)** Representative images of gross morphology from livers of WT (n=9) and Hpcal1-/- (n=7) mice. The tumor lesions are indicated by black arrows. **(C)** Analyses of the ratio of liver to body weight (left) and the number of tumors (right). **(D)** Representative images of hematoxylin and eosin (HE), HPCAL1 and Ki67 staining of livers from WT and Hpcal1-/- mice. scale bar, 50 µm. **(E)** ALT and AST levels in the sera of WT and Hpcal1-/- mice. AST, aspartate aminotransferase. ALT, alanine aminotransferase. **(F)** Schematic representation of constructs for MYC-Trp53 liver cancer model. **(G)** Scheme of MYC-Trp53 liver cancer model murine liver cancer model. hd.i, hydrodynamic injection. Mice were injected with 12 µg of MYC-Luc, 12 μg of sgp53, 12 µg of sgCON or sgHpcal1 and 3 µg of SB13 transposase-encoding plasmid mixed in 2 mL of 0.9% NaCl solution within 5 to 7 seconds. This figure was drawn by using BioRender (JX24G8PWZC) (H) Representative images of gross morphology from livers of sgCON (n=5) and sgHpcal (n=5) mice. **(I)** Analyses of the ratio of liver to body weight (left) and the number of tumors (right). **(J)** Representative images of HE, HPCAL1 and Ki67 staining of livers from sgCON and sgHpcal mice. scale bar, 50 µm. **(K)** Statistical analysis of the percentage of Ki67+ cells from indicated mice. **(L)** Kaplan-Meier survival curves of sgCON (n=8) and sgHpcal (n=8) mice. Log-rank Mantel-Cox test was used to calculate significance. Scale bar, 50 µm. Unpaired t test was used to determine statistical significance. *, **, *** means p < 0.05, p < 0.01, and p < 0.001.

To investigate the role of HPCAL1 in hepatocarcinogenesis of genetically engineered mouse models (GEMM), we prepared *Hpcal1* knockout (*Hpcal1*^-/-^) mice using CRISPR/Cas9-mediated gene editing to delete exon 2 and 3 of *Hpcal1* (Figure S1A). The *Hpcal1*-KO status was validated PCR using specific primers, gene sequencing and western blotting (Figure S1B-C). HCC mouse model was then established using diethylnitrosamine (DEN)/CCl4 consisting of the injection of 25 mg/kg of DEN, followed by intraperitoneal injection of CCL4 for 12 weeks as a tumor promoter, and the tumor burden was analyzed after 12 weeks (Figure 1A). Compared with the wide type counterparts (WT), *Hpcal1*^-/-^ mice had higher liver weight and liver-body ratio, and more tumor numbers (Figure 1B-C, Figure S1D-E). Consistently, we observed bigger histological lesions and enhanced proliferative activity (Ki67 reactivity) in the *Hpcal1*-deficient livers (Figure 1D and Figure S1F). Moreover, biochemical parameters such as alanine aminotransferase (ALT) and aspartate aminotransferase (AST) were significantly increased in serum of* Hpcal1*-deficient mice than those of WT (Figure 1E). These data indicate that *Hpcal1* loss promotes hepatocarcinogenesis in mice. To validate this hypothesis in liver-specific deletion of *Hpcal1* and established the clinical relevance between HPCAL1 and key driver genes of human HCC, we take advantage of hydrodynamic tail-vein injections to generate a MYC*/Trp53*^-/-^ liver cancer model in which oncogenic MYC can be genomically integrated, and that TP53 is deficient to recapitulate the features of HCC, as previously described 34. *Hpcal1* was genetically deleted using 4 sgRNA (small guide RNA), which was the same as those in *Hpcal1*^-/-^ mice (Figure 1F-G). The knockout efficiency was confirmed by western blotting (Figure S1G). As expected, liver-specific loss of *Hpcal1* significantly facilitated tumorigenesis with evidence of increased liver weight, liver-body ratio, tumor number and liver tumor cell proliferation activity (Figure 1H-1K and Figure S1H-1J). More importantly, *Hpcal1* loss in liver greatly shortened the overall survival times of mice compared to control group (Figure 1L). These data indicate that HPCAL1 deficiency in mice promotes hepatocarcinogenesis.

### *Hpcal1* loss increase lipid biosynthesis

**Figure 2:**
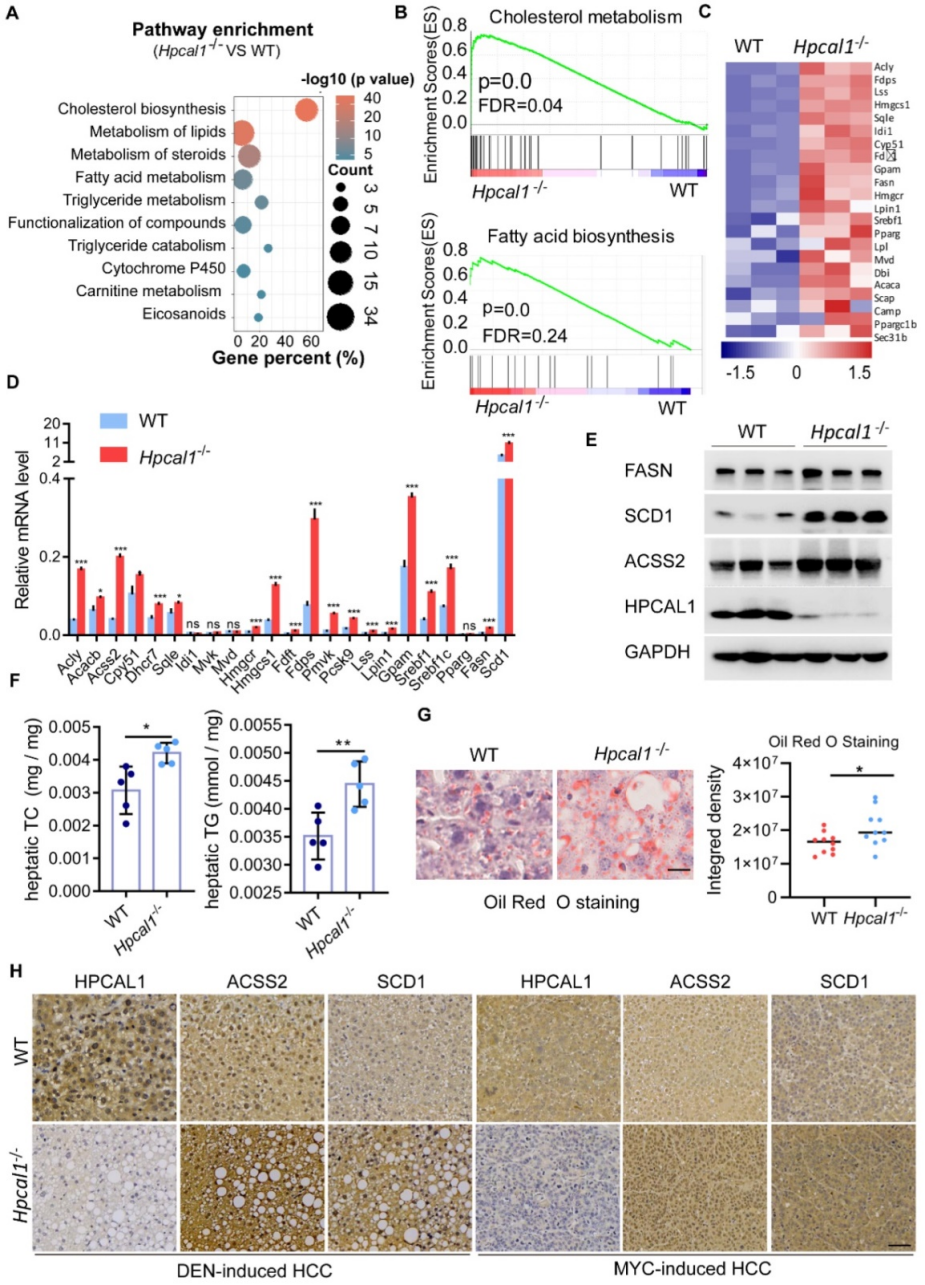
** Hpcal1 deletion promotes lipid metabolism in livers. (A)** Pathway enrichment analysis of the differential expression genes from the RNA-sequencing datasets of liver tumors of WT (n=3) and Hpcal1-/- (n=3) mice. **(B)** Gene set enrichment analysis (GSEA) showed enrichment of cholesterol and fatty acid biosynthesis in liver tumors of Hpcal1-/- (n=3) mice compared with WT mice. **(C)** Heatmap demonstration of the gene expression related to cholesterol and fatty acid biosynthesis. **(D)** Realtime PCR analysis of the mRNA levels of genes related to cholesterol and fatty acid biosynthesis. **(E)** Western blotting analysis of the cell lysates from liver tumors of WT and Hpcal1-/- mice with indicated antibodies. **(F)** Determination of hepatic total cholesterol (TG) and total triglyceride (TG) from liver tumors of WT (n=5) and Hpcal1-/- (n=5). **(G)** Representative images and statistical analysis of Oil Red O staining of liver sections from WT and Hpcal1-/-. Staining intensity of 2 fields per liver section from 5 mice of indicated group were calculated using Image J software. Scale bar, 25 µm.** (H)** Representative IHC images of HPCAL1, ACSS2 and SCD1 of liver sections from WT and Hpcal1-deficient mice. Unpaired t test was used to determine statistical significance. Scale bar, 50 µm. *, **, *** means p < 0.05, p < 0.01, and p < 0.001.

To explore the underlying mechanisms by which *Hpcal1* mediates tumorigenesis, we performed RNA-sequencing of liver tumor derived from WT and *Hpcal1*^-/-^ mice to identify signaling pathways potentially involved. By pathway enrichment analysis, it was found that lipid metabolic pathways, including cholesterol, fatty acids and steroids, were remarkably enriched upon *Hpcal1* loss in mice (Figure 2A), which was also supported by the result of gene set enrichment analysis (GSEA) (Figure 2B). The heat map of top upregulated gene implicated in cholesterol and fatty acids metabolism was shown (Figure 2C). The mRNA levels of selected 23 key genes implicated in lipid metabolism were further verified by real-time PCR. In line with the observation in RNA-seq datasets, these genes were remarkably upregulated in liver tumors of *Hpcal1*^-/-^ mice relative to WT tumors (Figure 2D). Furthermore, some key genes including* Fasn, Scd1* and* Acss2*, were examined by western blotting, which also showed higher protein abundance in *Hpcal1*-depleted tumors (Figure 2E). These evidences are supportive of overall upregulated genes in lipid biosynthesis. To confirm whether observed gene upregulation ultimately results in lipid biosynthesis in tumors upon *Hpcal1* deletion, we determined the hepatic levels of triglyceride and cholesterol. Indeed, higher levels of triglyceride and cholesterol were observed in *Hpcal1*-depleted tumors relative to WT (Figure 2F). This observation was supported by bigger and greater lipid droplets, evidenced by Oil Red O staining and visual estimation by phase contrast microscopy (Figure 2G). In addition, we found that *Hpcal1*-deficient liver tumors had much higher level of ACSS2 and SCD1 than WT tumors (Figure 2H and Figure S1K). Taken together, we demonstrate that *Hpcal1* loss in liver tumors enhances lipid biosynthesis.

### HPCAL1 directly interacts with RUVBL1/2 in an Ca^2+^ independent manner

**Figure 3:**
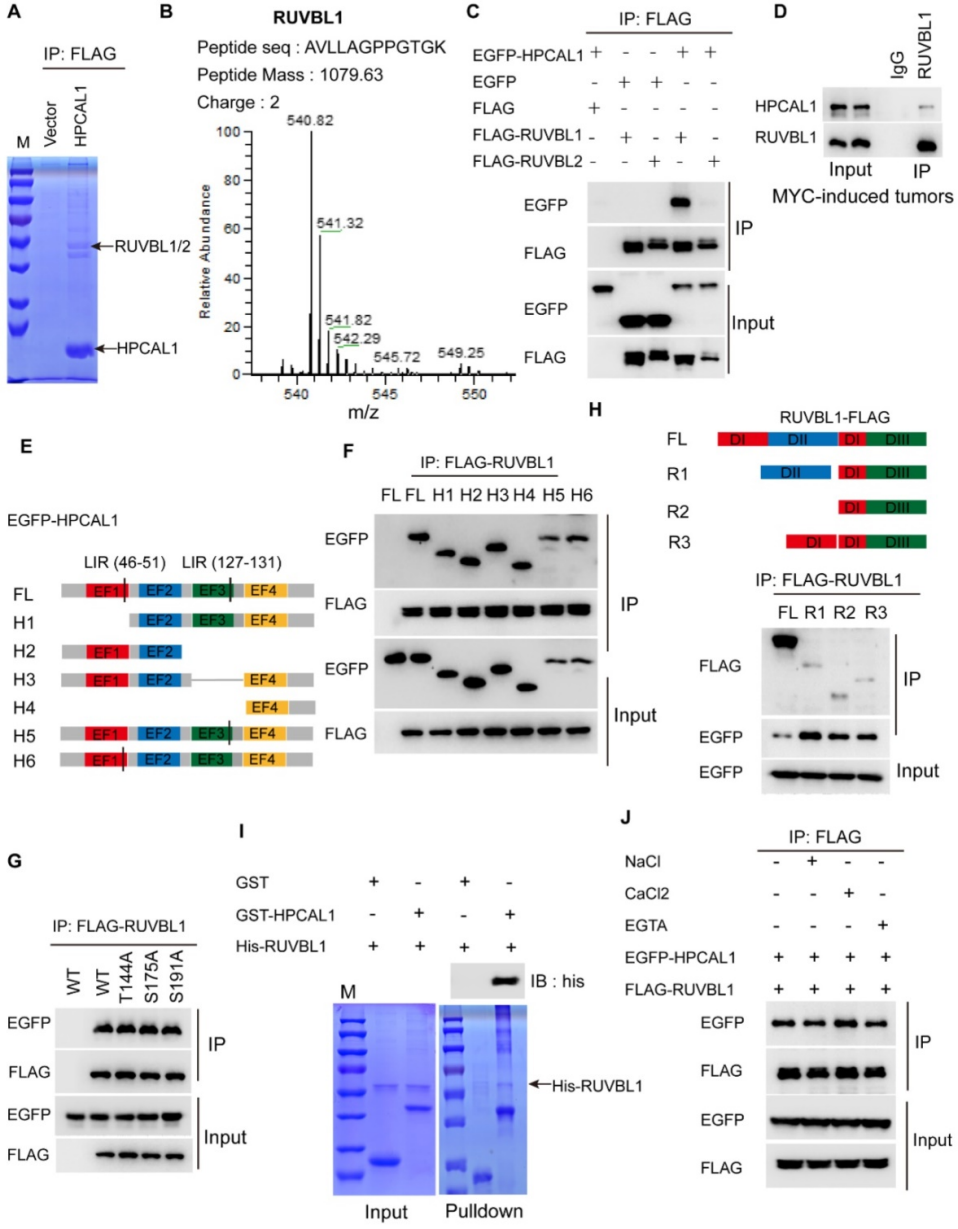
** HPCAL1 directly binds to RUVBL1. (A)** HEK293T transfected with FLAG-HPCAL1 or control plasmid were subjected to Co-IP/MS. Co-IP, co-immunoprecipitation. MS, mass spectrometry. **(B)** Protein information and representative mass spectrum peak of RUVBL1. **(C)** HEK293T cells were co-transfected with EGFP-HPCAL1 and FLAG-RUVBL1 or RUVBL2 plasmids. Immunoblot analysis of the cell lysates and immunoprecipitates with the indicated antibodies. **(D)** Endogenous HPCAL1 derived from MYC-induced liver tumors was immunoprecipitated using anti-HPCAL1 antibody or isotype IgG control and subjected to immunoblot analysis using indicated antibody. **(E)** Scheme of HPCAL1 domain organization. EF, EF-hand. **(F)** HEK293T cells were co-transfected with EGFP-HPCAL1 fragment and FLAG-RUVBL1 plasmids. Immunoblot analysis of the cell lysates and immunoprecipitates with the indicated antibodies. **(G)** HEK293T cells were co-transfected with various EGFP-HPCAL1 mutants and FLAG-RUVBL1 plasmids. Immunoblot analysis of the cell lysates and immunoprecipitates with the indicated antibodies. **(H)** Scheme of RUVBL1 domain structures and Co-IP assay. HEK293T cells were co-transfected with EGFP-HPCAL1 and various FLAG-RUVBL1 fragment plasmids. Immunoblot analysis of the cell lysates and immunoprecipitates with the indicated antibodies. DI, domain I; DII, domain II; DIII, domain III. **(I)** Purified recombinant GST-HPCAL1 was incubated with purified his-RUVBL1. Coomassie blue staining and immunoblot analysis of the cell lysates or immunoprecipitates with indicated antibody. **(J)** Co-IP and immunoblot analysis of RUVBL1/HPCAL1 interaction in the presence of NaCl (10 mM), CaCl2 (10 mM), and ETGA (10 mM).

To dissect the molecular basis of HPCAL1 in mediating lipid metabolism, we performed co-immunoprecipitation (Co-IP) and mass spectrometry (MS) to identify potential signaling molecules related to lipid biosynthesis (Figure 3A). Notably, RUVBL1/2, the AAA+ ATPases (ATPases associated with diverse cellular activities) that are in complex with TTT controlling mTOR assembly, activation and lipid metabolism 25, 39, was identified as the potential binding partners (Figure 3B, Figure S2A-B and Table S1). Co-IP assays showed that HPCAL1 indeed interacted with RUVBL1/2 in the HEK293T cells, and it appears that HPCAL1 bound to RUVBL1 with higher intensity relative to RUVBL2 (Figure 3C). Moreover, this interaction was further confirmed in the mouse liver tumors with endogenous proteins (Figure 3D). To better understand the nature of the RUVBL /HPCAL1 interaction, we defined the protein regions responsible for their reciprocal binding. To this end, serially truncated HPCAL1 or RUVBL1 motif sequence were prepared and co-transfected into HEK293T cells. It was shown that multiple domains of HPCAL1 likely mediated its interaction with RUVBL1 (Figure 3E and 3F). Also, mutation of several phosphorylation sites identified previously did not clearly affected HPCAL1/RUVBL1 interaction (Figure 3G), indicating that multiple domains of HPCAL1 likely bind to RUVBL1 independently. Likewise, it seems that all 3 domains of RUVBL1 were required for HPCAL1 binding as deletion of either one will affect the association (Figure 3H). In an effort to investigate whether this binding is direct, we prepared bacterial-expressed and purified HPCAL1 and RUVBL1 with GST and his tag, respectively. GST pulldown analysis revealed a direct binary association between HPCAL1 and RUVBL1 (Figure 3I). Furthermore, considering that HPCAL1 undergoes post-translational N-terminal myristylation, conformational change and membrane localization in response to cellular calcium (Ca^2+^) levels 40, we next test whether the interaction between HPCAL1 and RUVBL1 is Ca2^+^-dependent. Addition of either calcium chloride (CaCl_2_) or EGTA, which increases or decreases Ca^2+^ levels, respectively, did not clearly affect HPCAL1/RUVBL1 binding (Figure 3J). Thus, this interaction is independent of the Ca^2+^ concentration.

### HPCAL1 negatively regulates mTOR signaling in mice and human HCC specimens

**Figure 4:**
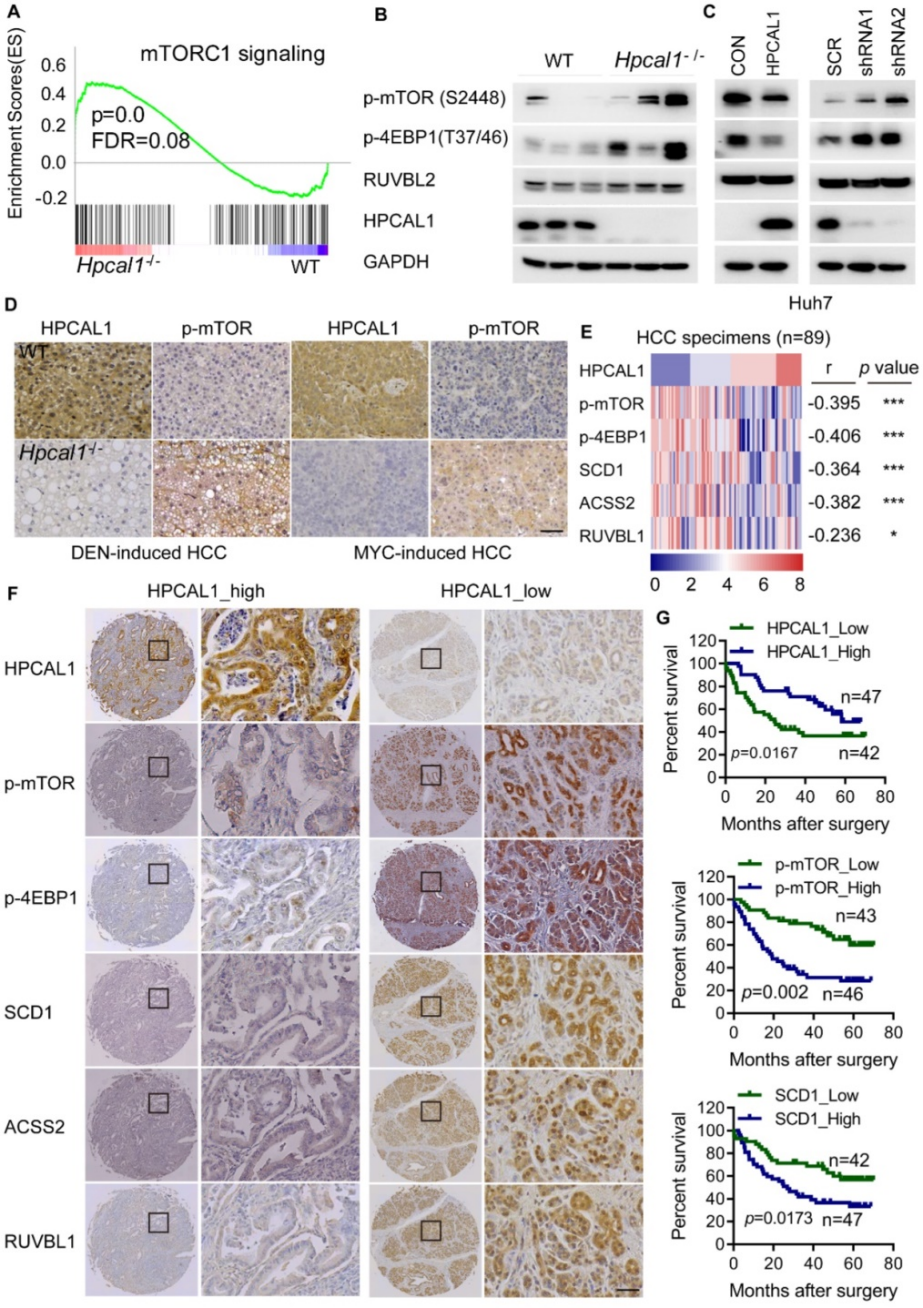
** HPCAL1 was inversely associated with mTOR signaling in mice and human. (A)** GSEA showed enrichment of mTOR signaling from the RNA-sequencing datasets of liver tumors of WT (n=3) and Hpcal1-/- (n=3) mice. **(B)** Western blotting analysis of the cell lysates from liver tumors of WT and Hpcal1-/- mice with indicated antibodies. **(C)** Immunoblot analysis of the cell lysates from Huh7 cells with HPCAL1 overexpression (left) or HPCAL1 depletion (right) using indicated antibodies. **(D)** Representative IHC images of HPCAL1 and p-mTOR of liver sections from WT and Hpcal1-deficient mice. **(E)** Heatmap demonstration of IHC score and correlation between PROX1, p-mTOR, p-4EBP1, SCD1, ACSS2 and RUVBL1 in HCC tissues (n=89). The criteria of IHC scoring were described in Materials and Methods. Pearson's correlation analysis was used to measure the relationship between indicated proteins in human HCC tissues. **(F)** Representative IHC images of indicated proteins derived from human HCC tissue microarray (TMA). **(G)** Kaplan-Meier curves analyses of patients with low versus high expression of indicated proteins HCC TMA. Scale bar, 50 µm.

Given that HPCAL1 directly binds to RUVBL1, which promotes mTOR signaling and lipid metabolism 41, 42, we reason that HPCAL1 may also regulate mTOR signaling via RUVBL1. Not surprisingly, genes related to mTOR signaling is significantly enriched in transcriptome datasets of liver tumors following Hpcal1 deficiency in mice, as revealed by GSEA (Figure 4A). This result was in agreement with observation that *Hpcal1*-depleted liver tumor showed higher phosphorylation levels of mTOR and its substrates 4EBP1 as well (Figure 4B). Moreover, HPCAL1 overexpression in Huh7 cells reduced mTOR activation, whereas depletion of HPCAL1 resulted in the opposite effect (Figure 4C). Consistently, IHC examination showed that the phosphor-mTOR levels were markedly higher in sections of *Hpcal1*-deleted liver tumors than their counterparts either initiated by DEN/CCL4 or MYC/sg*p53* (Figure 4D). To provide the clinical relevance between HPCAL1 and mTOR signaling, we investigated the expression correlation among HPCAL1, phosphor-mTOR, phosphor-4EBP1, SCD1, ACSS2 and RUVBL1 in the tissue microarray of 90 cases of HCC specimens (Table S2). Through IHC staining and grading, we found that HPCAL1 was negatively associated with the components of mTOR signaling and lipid metabolic enzymes (Figure 4E-F and Figure S3A-3B). Although HPCAL1 did not affect the expression of RUVBL1, the expression of these two proteins was also inversely correlated in HCC tissues. Furthermore, while low expression of HPCAL1 predicted unfavorable clinical outcome in patients with HCC, high expression of phosphor-mTOR, phosphor-4EBP1, SCD1, ACSS2 and RUVBL1 was correlated with worse survival times and (Figure 4G and Figure S3C-3E). Together, these results demonstrate that HPCAL1 represses mTOR signaling and lipid metabolism related to HCC progression and patient survival.

### HPCAL1 suppresses mTOR signaling and HCC progression via TTT-RUVBL1 assembly

**Figure 5:**
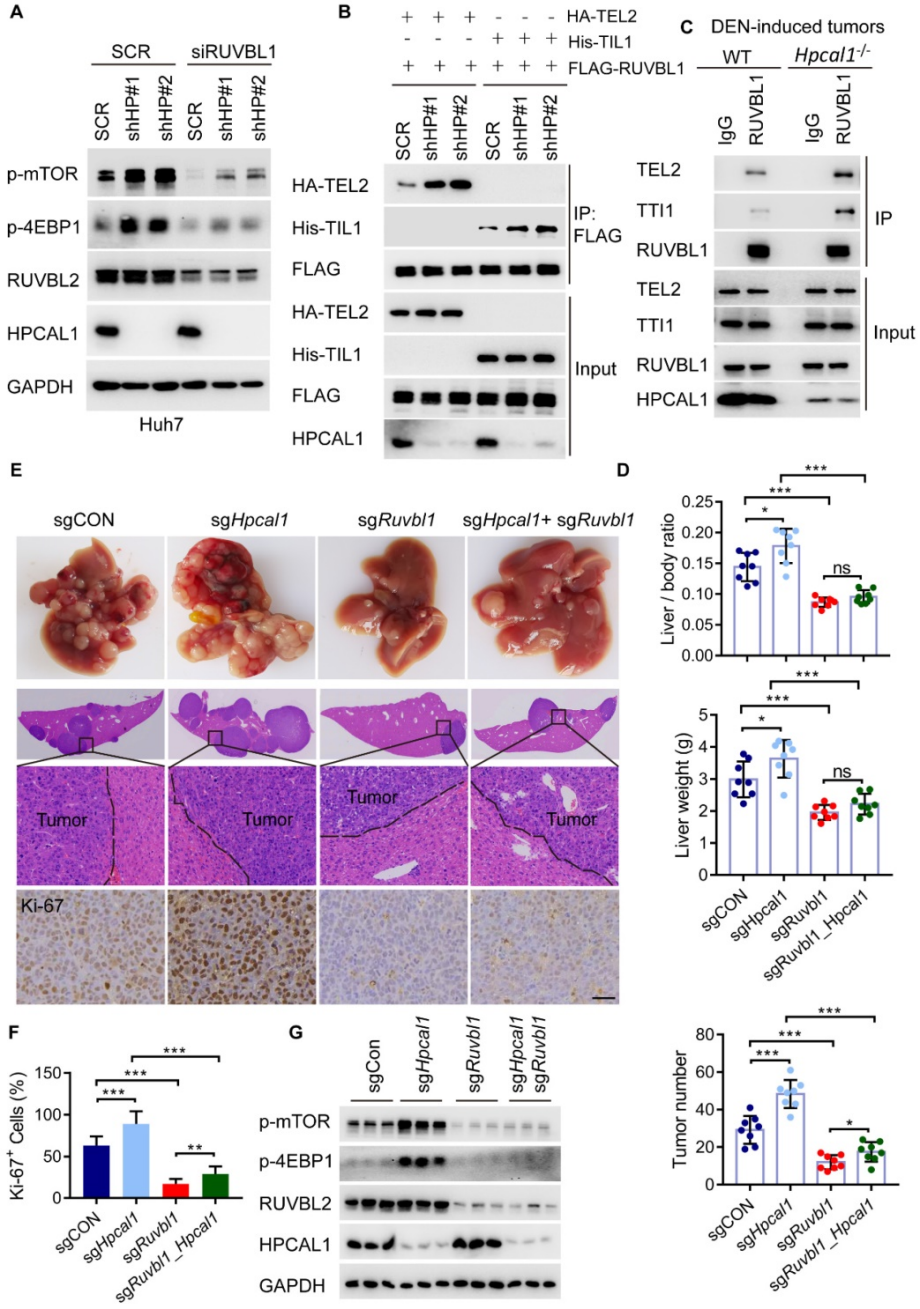
** HPCAL1 suppresses liver tumorigenesis via RUVBL1-dependent mTOR activation. (A)** Immunoblot analysis of the HPCAL1-depleted Huh7 cell lysates with or without RUVBL1 knockdown. HP, HPCAL1. **(B)** HEK293T cells co-transfected with FLAG-RUVBL1 and HA-TEL2 or his-TTI1 in the presence or absence of HPCAL1 were subjected to Co-IP and immunoblot analysis with indicated antibodies. **(C)** Co-IP and immunoblot analysis of the lysates of liver tumors from WT and Hpcal1-/-. **(D)** Statistical analyses of the ratio of liver to body weight (upper), liver weight (middle) and the tumor number (lower) from livers of indicated mice (n-=8). **(E)** Representative images of gross morphology, HE and IHC staining from liver section of indicated mice. **(F)** Statistical analysis of the percentage of Ki67+ cells indicated mice. **(G)** Immunoblot analysis of the lysates of liver tumors from indicated mice using indicated antibodies. Scale bar, 50 µm.

To clarify whether HPCAL1 regulates mTOR signaling via its binding to RUVBL1, we investigate mTOR activity in Huh7 cells with simultaneous depletion of HPCAL1 and RUVBL1/2. As showed, HPCAL1 ablation significantly increased levels of mTOR and 4EBP1 phosphorylation, which was largely abrogated in Huh7 cells following RUBVBL1/2 knockdown (Figure 5A). Given that RUVBL1/2 in complex with TTT is required for mTOR activation, we next examine whether the levels of HPCAL1 would affect TTT-RUVBL1 complex formation. Expectedly, the association between RUVBL1 and TEL2 or TTI1, two essential components of TTT complex, was greatly increased in Huh7 cells upon HPCAL1 depletion relative to control cells (Figure 5B). Using samples derived from DEN-induced liver tumors, we further showed a similar effect in liver tumors (Figure 5C), indicating that HPCAL1 indeed affects TTT-RUVBL1 complex, thus mTOR activation. We next ask whether the observed signaling event would ultimately alter HCC progression *in vivo*. To this end, we used a MYC*/Trp53*^-/-^ liver cancer model and CRISPR/Cas9 to delete individual *Hpcal1* and *Ruvbl1* or both in mouse livers. *Ruvbl1* loss alone led to significant repression of liver tumorigenesis, as previously reported 41. Consistent with the observation that *Hpcal1* deficiency resulted in dramatical increase of tumor growth above, this effect was partially reversed in tumors with simultaneous deficiency of *Hpcal1* and *Ruvbl1* (Figure 5D-E), considering that the tumor number was higher compared with that of *Ruvbl1*-deleted tumors. Histological analyses confirmed the knockout efficiency and the consistent effect with evidence of Ki-67 reactivity (Figure 5F and Figure S4). We further performed western blotting demonstrating that mTOR activation was significantly compromised in *Ruvbl1-*deleted and or co-deleted tumors (Figure 5G). These results suggest that HPCAL1 blunts liver tumor growth partly through RUVBL1/2-mediated mTOR activation.

### HPCAL1 renders liver tumors resistant to mTORi

**Figure 6:**
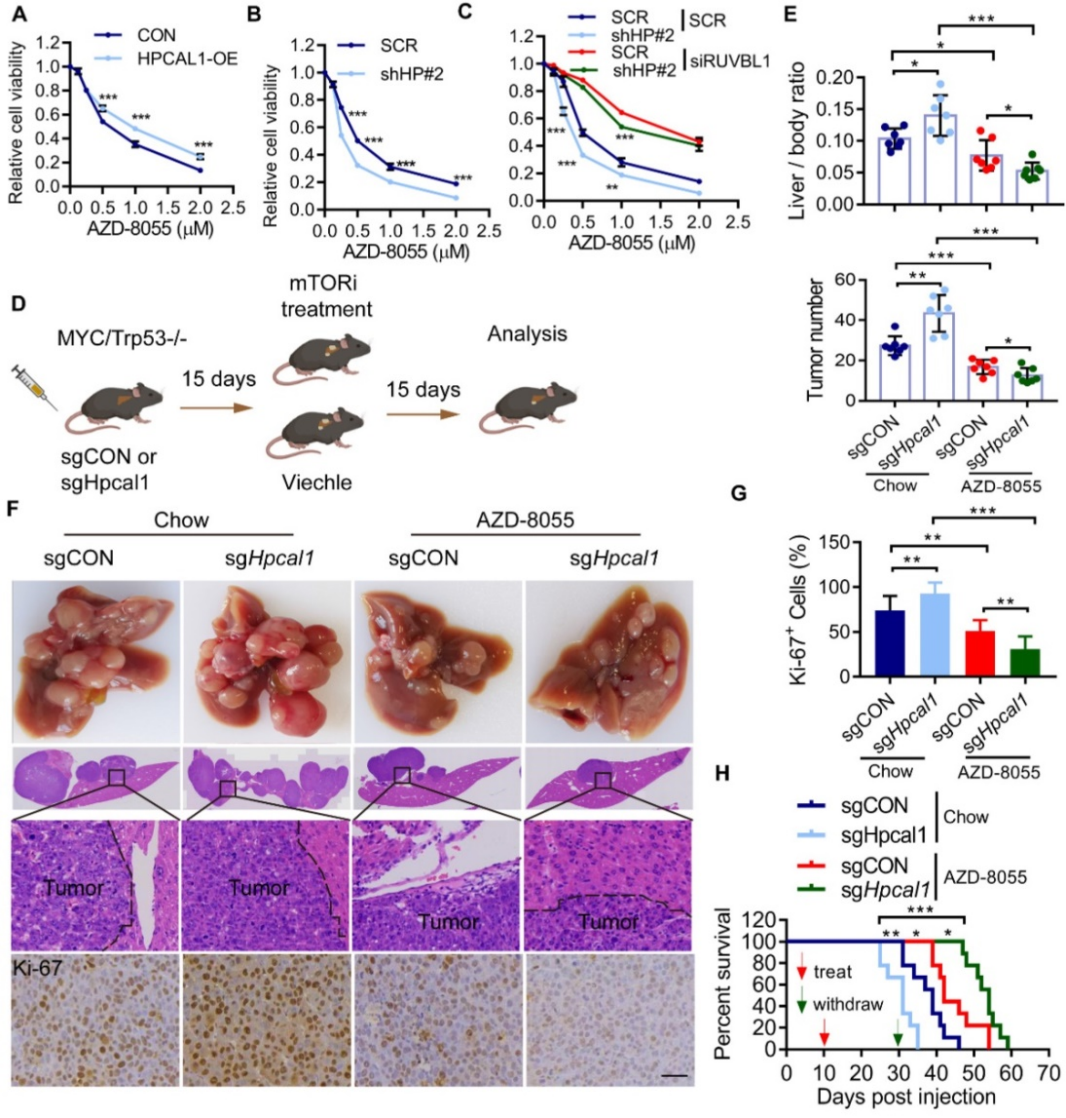
** HPCAL1 suppresses liver tumorigenesis via RUVBL1-dependent mTOR activation.** Short-term growth inhibition assay of HPCAL1-overexpressed **(A)** or HPCAL1-depleted **(B)** Huh7 cells treated with indicated concentration of AZD-8055. OE, overexpression. HP, HPCAL1. **(C)** Short-term growth inhibition assay of SCR or HPCAL1-depleted Huh7 cells transfected with SCR or siRUVBL1 and treated with indicated concentration of AZD-8055. **(D)** Schematic representation of MYC-Trp53 liver cancer model and mTORi treatment. This figure was drawn by using BioRender (JX24G8PWZC) **(E)** Statistical analyses of the ratio of liver to body weight (upper) and the number of tumors (lower) from livers of indicated mice fed with regular chow or chow with additional AZD-8055 at the dose of 67 mg/kg (n=7). **(F)** Representative images of gross morphology, HE and IHC staining from liver section of indicated mice. **(H)** Statistical analysis of the percentage of Ki67+ cells from indicated mice. **(G)** Kaplan-Meier curves analyses of indicated mice with or with dietary addition of AZD-8055 (n=9). Scale bar, 50 µm. *, **, *** means p < 0.05, p < 0.01, and p < 0.001.

In an effort to exploit the therapeutic opportunity based on the observed mechanism, we test whether HPCAL1 would affect the sensitivity of mTORi such as AZD8055, a potent, selective, and orally bioavailable ATP-competitive mTOR kinase inhibitor 43. As expected, while HPCAL1 overexpression blunts the response of Huh7 cells to AZD8055, HPCAL1 depletion increased the sensitivity of Huh7 cells to AZD8055, as evidenced by CCK8 assay (Figure 6A, 6B and Figure S5A). This role of HPCAL1 was demonstrated to be dependent on RUVBL1/2 (Figure 6C). Similar results were confirmed in Huh7 cells with colony formation assay (Figure S5B-D). To extend this observation in mouse model, control and *Hpcal1*-depleted tumors in mice were treated with or without AZD8055. Strikingly, compared with control tumors, although *Hpcal1*-depleted mice had larger tumors, they showed higher sensitivity to AZD8055, with lower liver weight, liver-body ratio, tumor numbers and diminished Ki-67 staining (Figure 6D-G and Figure S5E-H). While the overall survival times was remarkably decreased in sg*Hpcal1* mice relative to sgCON mice fed with chow, it was greatly prolonged in mice deficient for *Hpcal1* following AZD-8055 treatment, although comparable reduction of mTOR and 4EBP1 phosphorylation was observed in tumors following AZD-8055 treatment (Figure 6H and Figure S5I). Together, we conclude that HPCAL is a novel negative regulator for liver lipid metabolism and *Hpcal1* deficiency renders liver tumors addicted to mTOR signaling and boosts the therapeutic response to mTOR inhibitor via RUVBL1/2-dependent mTOR assembly.

## Discussion

The EF hand domain, which sequesters Ca^2^ professionally, is one of the most common domains encoded by the human genome, in contrast with a poor knowledge of their physiological functions 31, 44, 45. We previously have identified that HPCAL1, a neural calcium sensor protein with four EF hand domains, was often lost in patients with HCC and correlated with deregulated cell cycle progression and an unfavorable prognosis 31. In this study, we have used DEN/CCl4 and MYC/Trp53-induced liver cancer model and demonstrated that genetic loss of HPCAL1 resulted in significantly enhanced liver tumorigenesis in mice, reinforcing the notion that HPCAL1 functions as a potential tumor suppressor in HCC. Although our prior work has indicated a role of HPCAL1 in cell cycle arrest via a direct binding to p21^WAF1/Cip1^ (ref), a potent cyclin-dependent kinase (CDK) inhibitor 31, its functions in HCC has been largely unknown. Intriguingly, we found here that *Hpcal1*-deficient tumors displayed molecular signature of lipid metabolic reprogramming and triggered numerous genes implicated in *de novo* fatty acid and cholesterol biosynthesis, an emerging hallmark of cancer metabolism occurs in aggressive tumors and correlates with therapeutic resistance 8, 12. Such deregulation of lipid metabolism was further linked to mTOR hyperactivation, which critically regulates many essential biological functions, including lipid metabolism. Paradoxically, tumors deficient for *Hpcal1* elicits hypersensitivity to mTORi AZD-8055, indicating that genetic inactivation of Hpcal1 in hepatocytes results in lipid metabolic changes that are critical for HCC progression and also a metabolic vulnerability that offer therapeutic opportunity to mTORi. This observation provides the rationale for HPCAL1 as a surrogate biomarker for HCC treatment with mTOR hyperactivation using mTORi, considering the frequent loss of HPCAL1 in HCC 31. Given that numerous mTORi, including rapamycin analogs and ATP-competitive mTOR inhibitors, have been approved and more mTORi are being evaluated in the clinical trials 46, it is not yet defined whether *Hpcal1*-deficient tumors respond similarly to these mTORi, and which is more effective. It should be noted that while many oncogene-addicted cancers, including mTOR, show striking initial responses to acute targeted therapies, most patients ultimately develop resistance being targeted 47-49. As such, this common pattern of resistance to mTOR-addicted therapy requires additional target(s) for combined therapy 50, 51, which might be interesting works in the future. Nevertheless, our data here establishes a functional link between HPCAL1 and therapeutic response via mTOR signaling.

Mechanistically, we uncovered that HPCAL1 directly bound to RUVBL1, inhibiting the assembly of TTT complex and mTOR activation. Such interaction occurs not only in cells, but also are present in established tumors of mice, underlying the physical importance of RUVBL1-HPCAL1 interaction in liver cancers. In agreement with the previous observations by us and others 31, 52, despite being as a Ca^2+^ sensor, HPCAL1 interacted with RUVBL1 that was clearly not affected by Ca^2+^ concentration in cancers.

Of note, RUVBL2, the additional essential component that belongs to AAA+ ATPase superfamily, appears to interact with HPCAL1 with lower intensity. However, depleting RUVBL1 alone was sufficient to reverse the tumorigenesis in *Hpcal1*-deficient mice, suggesting HPCAL1 may function that is largely dependent on the intact hetero-hexamer of RUVBL proteins 28, 39, 53. As the TTT- RUVBL1/2 complex is required for the functional assembly of mTOR and activation 27, the observation that HPCAL1 deficiency in HCC cells and tumors as well facilitates the binding of RUVBL1 to TEL2 and TTI1, two essential components of TTT complex, solidifies that HPCAL1 is indeed implicated in the TTT-RUVBL1/2 complex formation. Although RUVBL1 depletion largely overcomes HPCAL1-mediated mTOR sensitivity and tumor progression, it is currently unknown whether there is other mechanism(s) potential involved, such as TSC1/2, AMPK and Rag upon energy stress 23. Since the mTOR activation respond to energetic stresses 13, such as glucose and amino acids, we did not observe a change in HPCAL1 expression in HCC cells and mice in response to glucose deprivation or glutamine (data not shown). Also, in liver tumors, which is exposed to glucose deficiency, Hpcal1 deletion boosted TTT- RUVBL1/2 complex formation. These lines of evidence suggested that HPCAL1 senses energetic signals to mTOR signaling that is irrelevant of its protein abundance, implying a potential post-translational modification(s) potential involved, which warrants future investigation. Clinically, we uncovered a negative association between HPCAL1 and mTOR signaling and lipid metabolic enzymes in human liver cancers. Notably, HPCAL1 expression is not significantly altered in livers of mice with HFD or type 2 diabetes (T2D) (data not shown), and we have not observed the clinical relevance of HPCAL1 in non-alcoholic fatty liver disease (NAFLD) and NAFLD-associated HCC in patients, due to limited clinical information available for relevant analysis. Given that NAFLD is increasingly ranking the top risk factor for human liver cancer 54, dissecting the role and clinical relevance of HPCAL1 with NAFLD-associated HCC is of great significance for potential cancer subtyping and therapy.

While the role of HPCAL1 in HCC growth and therapeutic response has been investigated here, it is current unknown and will be an interesting work in future whether HPCAL1 is implicated in the aggressive phenotypes of HCC. In addition, although we used two separate HCC mouse model, the observations and conclusions obtained from the whole-body knockout mice did not indicate a direct role of HPCAL1 in livers. We can't rule out the possibility of potential side effect derived from other organs upon HPCAL1 deficiency, particularly considering that HPCAL1 is a neural calcium sensor that is expressed at higher levels in brain than liver (Human Protein Atlas). Nevertheless, this is an unresolved and interesting work in the future using conditional-knockout mouse model. Collectively, this study identified HPCAL1 as an important regulator of lipid biosynthesis and mTOR addiction, and proposed a metabolic vulnerability in Hpcal1-deficient HCC that could be exploited for antitumoral therapy using mTORi.

## Supplementary Material

Supplementary figures and tables.

## Abbreviations

HPCAL1: Hippocalcin-Like 1
RUVBL1/2: RuvB Like AAA ATPase 1/2
mTOR: Mammalian target of rapamycin
TEL2: Telomere length regulation protein TEL2 homolog
TTI1/2: TELO2-interacting protein 1 homolog 1/2
RHEB: Ras homologue enriched in brain
TSC1/2: the tuberous sclerosis heterodimer 1/2
4EBP1: eukaryotic translation initiation factor 4E-binding protein 1
HE: hematoxylin and eosin staining
IHC: immunohistochemical
CCK8: Cell Counting Kit 8
