# Supplementary material for: Hippocalcin-Like 1 blunts liver lipid metabolism to suppress tumorigenesis via directly targeting RUVBL1-mTOR signaling
Source: Theranostics. 2022 Oct 24;12(17):7450–64. doi: 10.7150/thno.75936 (PMC9691343; doi:10.7150/thno.75936)
Supplement: Supplementary file 1 — Supplementary figures and tables. [file thnov12p7450s1.pdf]

Supplemental Figure legend

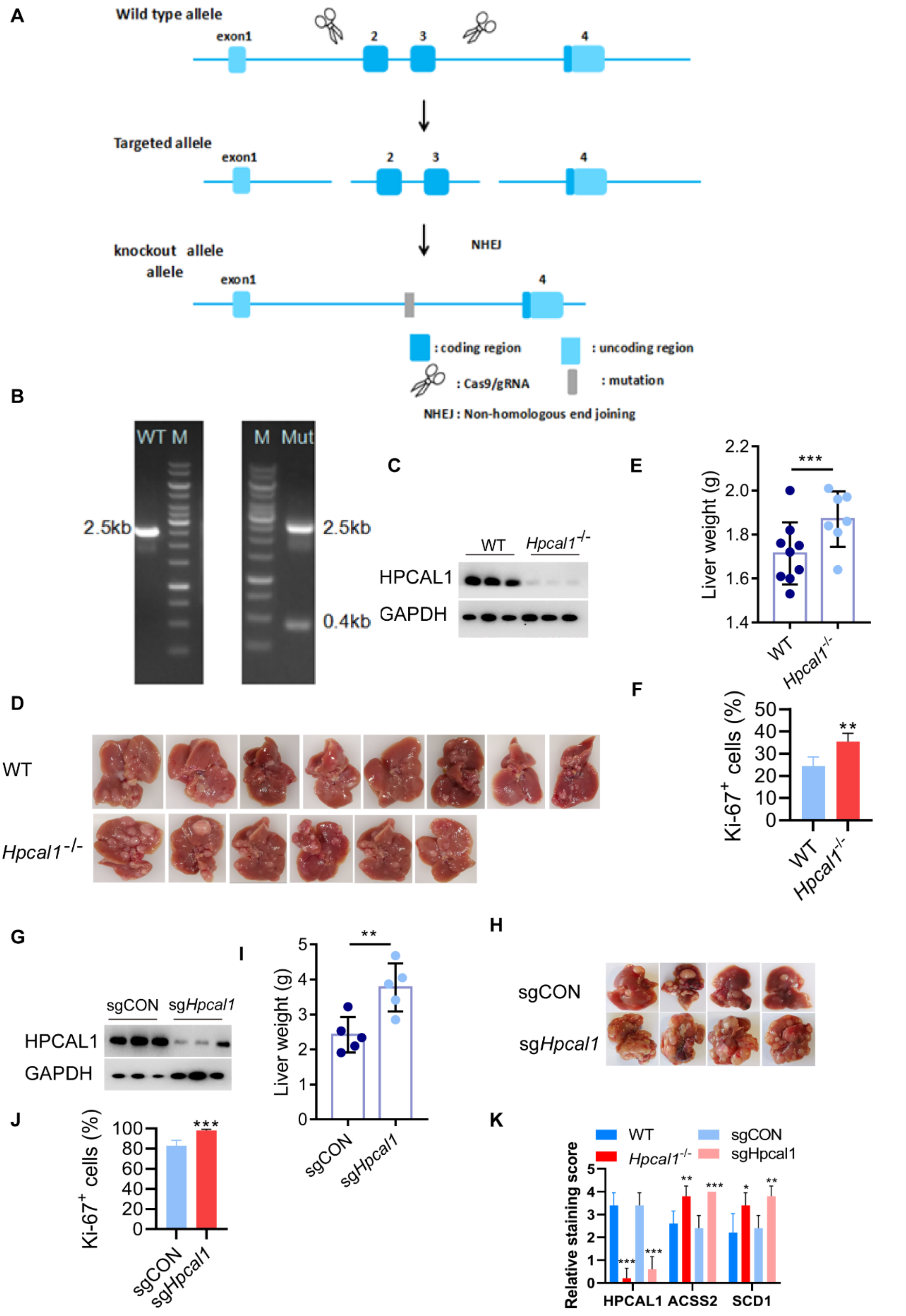

Supplemental Figure 1. Strategy for Hpcal1 knockout in mice using CRISPR–Cas9 system.

(A) Schematic representation of the targeting construct using the CRISPR–Cas9 system, resulting in *Hpcal1*$^{-/-}$ mice; sgRNA, single guide RNA. (B) Representative images of agarose gel electrophoresis of PCR products in WT and *Hpcal1*$^{-/-}$ mice. (C) Immunoblot verification for *Hpcal1* knockout efficiency using the lysates of liver tumors from WT and *Hpcal1*-deficient mice of DEN/CCL4. (D) Gross images of livers from indicated mice. (E) Statistical analysis of liver weight in sgCON and sg*Hpcal1* mice. (F) Statistical analysis of the percentage of Ki67^{+} cells indicated mice. (G) Immunoblot verification for *Hpcal1* knockout efficiency using the lysates of liver tumors from WT and *Hpcal1*$^{-/-}$ mice. (H) Gross images of livers from indicated mice. (I) Statistical analysis of liver weight in sgCON and sg*Hpcal1* mice. (J) Statistical analysis of the percentage of Ki67^{+} cells indicated mice. (K) Statistical analysis of IHC staining intensity of indicated proteins in indicated mice.

A

Protein name	Unique peptide
RUVBL2	12
RUVBL1	7

B

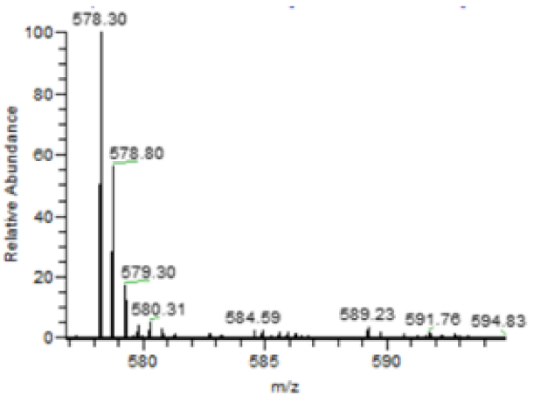

C

D

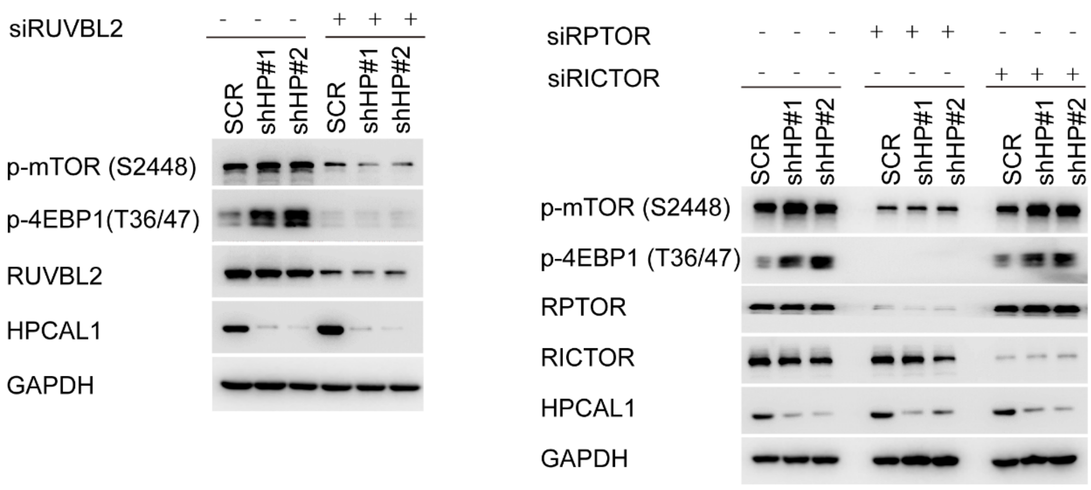

Supplemental Figure 2. LC-MS/MS identification of potential interactors.

(A) Summary of RUVBL1 and RUVBL2 protein identification. (B) This mass spectrum of representative peptide of RUVBL1. (C) Immunoblot analysis of the HPCAL1-depleted Huh7 cell lysates with or without RUVBL1 knockdown using indicated antibodies. (D) Immunoblot analysis of the HPCAL1-depleted Huh7 cell lysates with or without RAPTOR or RICTOR knockdown using indicated antibodies.

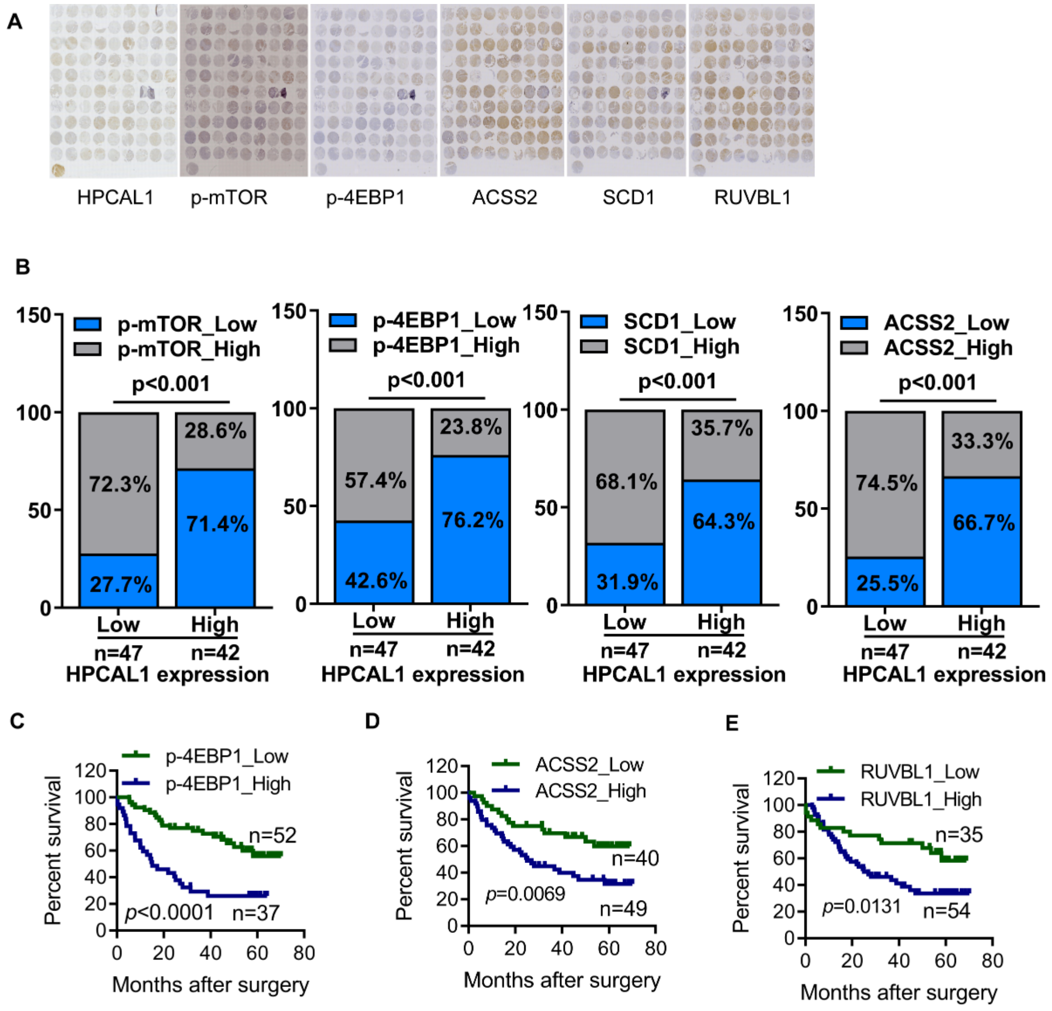

Supplemental Figure 3. 4EBP1 and its downstream targets predict unfavorable prognosis in patients with HCC.

(A) IHC images of HCC tissue microarrays indicated antibodies. (B) Correlation analysis of HPCAL1 with p-mTOR, p-4EBP1, SCD1 and ACSS2 using two-sided Chi-square test. Kaplan-Meier curves analyses of patients with low versus high expression of p-4EBP1 (C) and ACSS2 (D) and RUVBL1 (E).

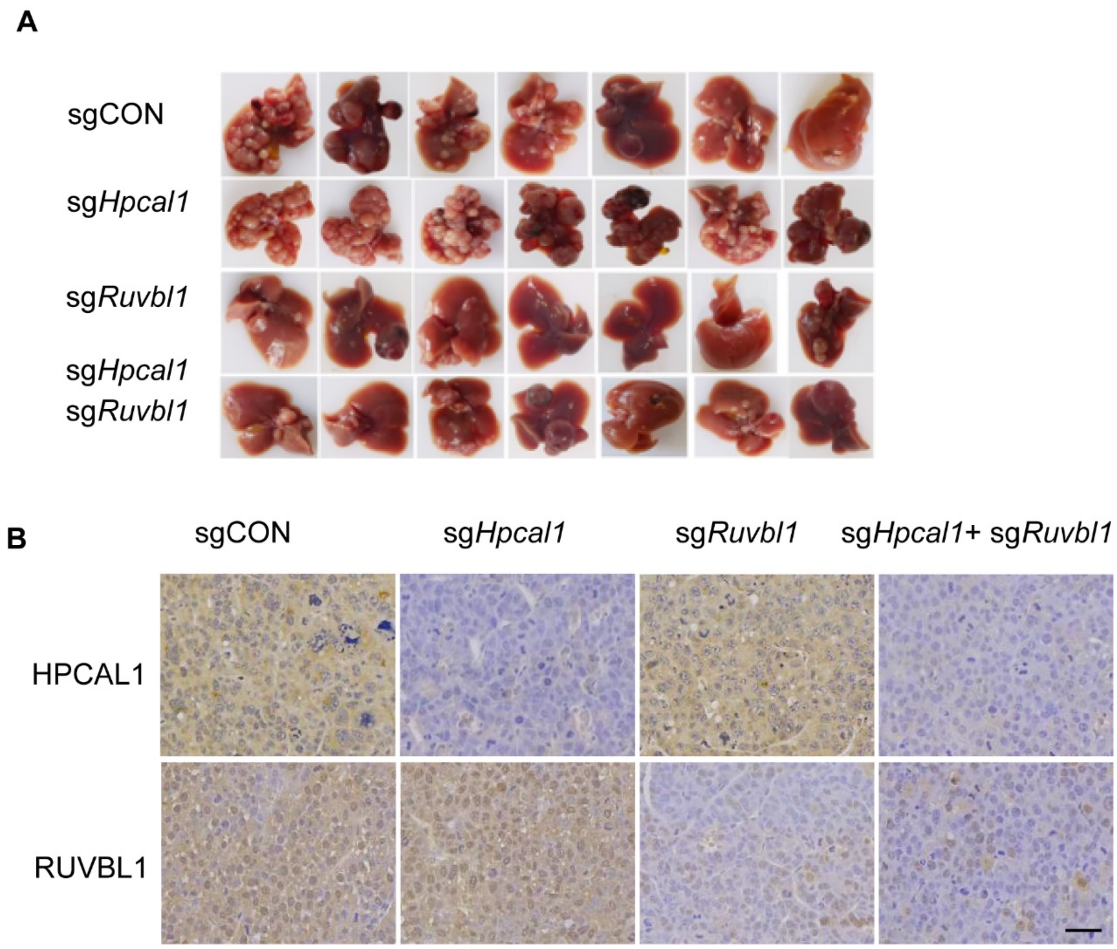

Supplemental Figure 4. Gross tumor images and IHC staining of Hpcal1 or Ruvbl1 knockout efficiency in mice livers.

(A) Gross images of livers from indicated mice. (B) IHC staining of *Hpcal1* and *Ruvbl1* in liver tumors of indicated mice. Scale bar, 50 µm.

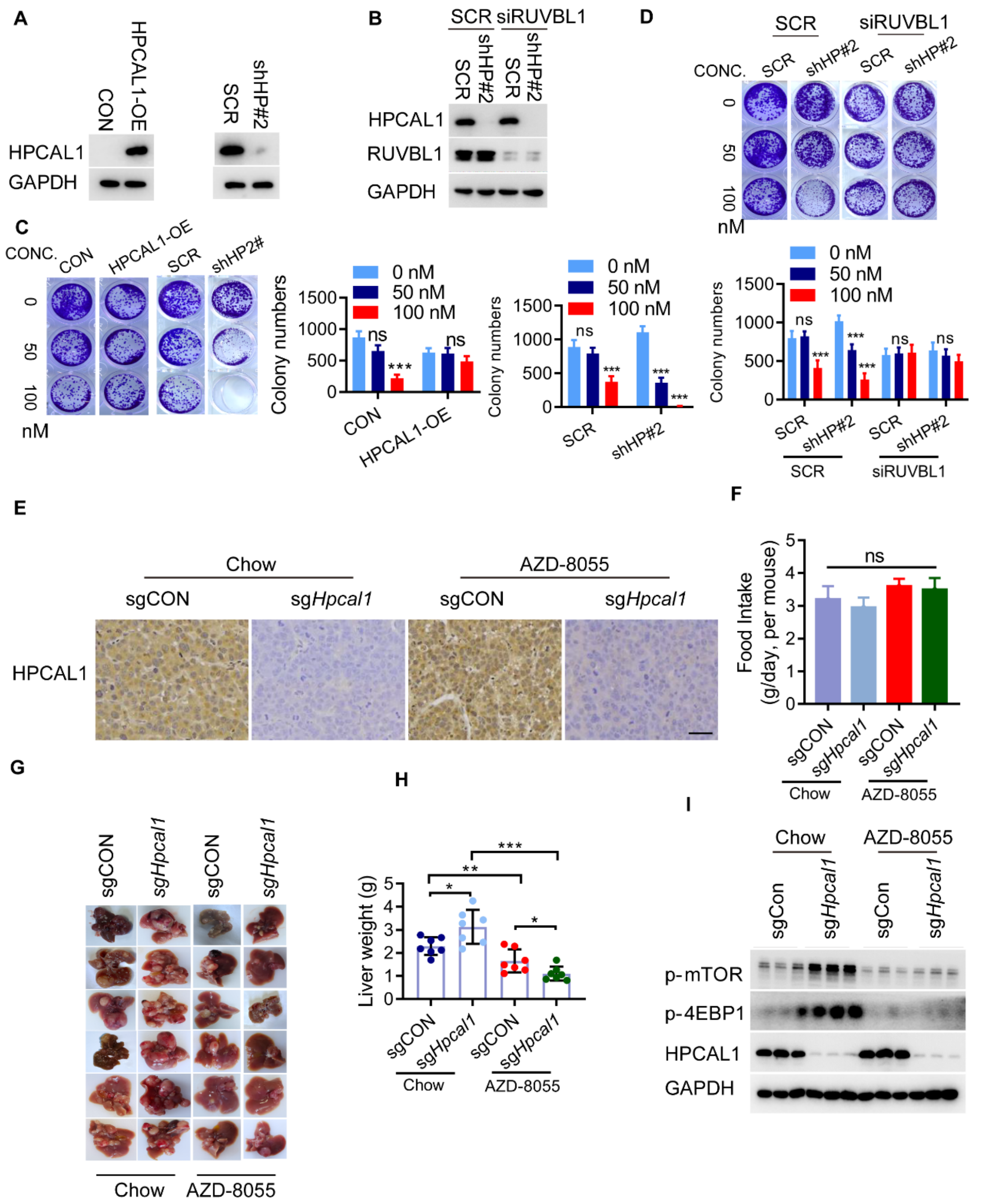

Supplemental Figure 5. RUVBL1 is indispensable for HPCAL1-mediated sensitivity of Huh7 cells to AZD-8055

(A) Immunoblot verification of the knockdown efficiency and overexpression of HPCAL1 in Huh7 cells. OE, overexpression. HP, HPCAL1. (B) Immunoblot verification of the knockdown efficiency of HPCAL1 and RUVBL1. (C) Huh7 cells infected with indicated lentivirus were seeded and grown in the presence of AZD-8055 at the indicated concentration for 10–14 days. Statistical analyses were shown. (D) Huh7 cells

infected with SCR or shHP#2 were transfected with SCR or siRUVBL1, followed by growth in the presence of AZD-8055 at the indicated concentration for 10–14 days. Statistical analyses were shown. SCR, scramble. HP, HPCAL1. (E) Verification of *Hpcal1* knockout in mice using IHC staining. (F) The food intake of indicated mice. (G) Gross images of livers from indicated mice. (H) Statistical analysis of liver weight from indicated mice. (I) Immunoblot analysis of the lysates of liver tumors from indicated mice with or with dietary addition of AZD-8055 (67 mg/kg). Scale bar, 50 µm.

Supplemental Table 1. Summary of the clinical information for patients with HCC

**Characteristics**	**Patients**
**Age, years**	
Median	62
Range	33-83
**Sex**	
Male	71
Female	18
**Etiology**	
Hepatitis B	na
Hepatitis C	na
**NAFLD& NASH**	
Yes	5
no	84
**Cirrhosis**	
Yes	44
no	45
**T**	
0-2	38
3-4	51
**N**	
Yes	1
no	88
**M**	
Yes	2
no	87
**Tumor size**	
<4cm	20
>4cm	69
**Survival Status**	
Yes	42
no	47

Supplemental Table 2. List of potential interacting partners of HPCAL1 by LC-MS/MS

Uniprot Accession	Protein name	Con (#Unique peptide)	HPCAL1 (#Unique peptide)	Con (coverage %)	HPCAL1 (coverage %)
P13639	EEF2	5	31	5.48	31.7
P11021	HSPA5	10	29	19.27	47.55
P07355	ANXA2	11	25	28.61	59
P15924	DSP	3	23	0.84	7.56
P38646	HSPA9	10	22	13.4	34.32
P14923	JUP	6	18	7.25	24.97
P10809	HSPD1	4	17	6.46	25.13
P49411	TUFM	5	15	10.62	36.73
Q07065	CKAP4	0	15	0	27.08
P37235	HPCAL1	4	14	20.73	55.44
Q9Y230	RUVBL2	2	14	5.4	29.81
P68104	EEF1A1	9	14	16.23	31.17
Q02413	DSG1	2	13	2.29	12.87
P04406	GAPDH	8	13	20.9	37.61
O00571	DDX3X	1	13	1.81	18.43
P30419	NMT1	0	12	0	24.6
Q92841	DDX17	0	12	0	18.93
Q9UGP8	SEC63	0	11	0	15
P30740	SERPINB1	2	11	5.28	27.18
P22234	PAICS	1	10	1.65	17.88
Q96JJ7	TMX3	0	10	0	17.84
P25705	ATP5F1A	4	10	7.05	17.18
Q9UBS4	DNAJB11	7	10	16.76	24.02
O15228	GNPAT	1	10	1.18	13.09
P49915	GMPS	0	10	0	14.29
P52272	HNRNPM	9	10	7.95	11.92
P50991	CCT4	1	10	1.3	18.18
P31153	MAT2A	3	10	6.84	25.32
P49368	CCT3	2	9	3.85	14.86
O75688	PPM1B	18	9	43.22	26.51
P31689	DNAJA1	4	9	10.08	25.69
P08195	SLC3A2	0	9	0	13.65
A6NCL7	ANKRD33B	0	9	0	20.85
Q96KC8	DNAJC1	0	9	0	19.13
Q6ZRP7	QSOX2	0	9	0	14.04
Q8IXB1	DNAJC10	7	9	7.44	10.34
P07948	LYN	4	8	12.3	21.09
P41250	GARS	0	8	0	9.74

P63151	PPP2R2A	0	8	0	17
Q9Y265	RUVBL1	2	8	5.04	18.2
P04843	RPN1	1	8	1.65	14.17
Q96A33	CCDC47	0	8	0	11.18
P36957	DLST	0	8	0	15.45
P17987	TCP1	1	7	2.16	12.41
P50990	CCT8	2	7	4.38	13.32
Q92945	KHSRP	5	7	7.88	13.22
O43175	PHGDH	3	7	5.82	14.07
P51648	ALDH3A2	0	7	0	15.46
Q8N2K0	ABHD12	0	7	0	13.57
Q9P258	RCC2	0	7	0	14.37
Q08188	TGM3	3	7	5.63	9.52
P06576	ATP5F1B	0	7	0	15.12
P25311	AZGP1	3	7	12.42	25.17
P09543	CNP	0	7	0	18.29
O60716	CTNND1	0	7	0	5.89
O60551	NMT2	0	7	0	15.66
Q9ULC5	ACSL5	1	7	1.17	10.1
Q9Y266	NUDC	0	6	0	22.66
P07947	YES1	5	6	11.23	13.63
P51570	GALK1	0	6	0	16.07
Q08554	DSC1	0	6	0	8.28
Q13308	PTK7	0	6	0	6.45
O60701	UGDH	0	6	0	13.56
Q16576	RBBP7	0	6	0	11.76
P28331	NDUFS1	2	6	3.03	10.32
Q8NC51	SERBP1	0	6	0	16.42
P14618	PKM	1	6	2.07	14.69
P33993	MCM7	0	6	0	8.62
O95573	ACSL3	0	6	0	8.33
Q9NTJ5	SACM1L	0	6	0	9.54
O75534	CSDE1	0	6	0	6.02
P0CG48	UBC	2	5	2.63	4.53
P04899	GNAI2	0	5	0	26.76
O43143	DHX15	0	5	0	5.79
P52292	KPNA2	1	5	1.7	12.48
P0CG47	UBB	2	5	7.86	13.54
Q01813	PFKP	0	5	0	5.1
P62979	RPS27A	2	5	11.54	19.87
P62987	UBA52	2	5	14.06	24.22
P29508	SERPINB3	1	5	3.08	10.77
P31943	HNRNPH1	4	5	12.47	10.24

Q99832	CCT7	1	5	1.29	9.02
P19474	TRIM21	4	5	8.84	10.32
Q6ZMU5	TRIM72	0	5	0	8.39
Q08209	PPP3CA	0	5	0	9.21
Q5VV42	CDKAL1	0	5	0	8.29
Q9NPH2	ISYNA1	0	5	0	9.32
Q14344	GNA13	0	5	0	20.69
O43684	BUB3	0	5	0	20.73
P22735	TGM1	1	5	1.1	7.22
Q14315	FLNC	0	5	0	2.17
P35813	PPM1A	0	5	0	21.47
P11413	G6PD	0	4	0	7.18
P13995	MTHFD2	0	4	0	11.71
P00390	GSR	3	4	5.75	7.09
P78371	CCT2	0	4	0	6.73
O43813	LANCL1	0	4	0	9.77
P56192	MARS	0	4	0	6
P31040	SDHA	1	4	1.66	5.87
P30153	PPP2R1A	0	4	0	7.81
O75342	ALOX12B	0	4	0	5.99
Q53H12	AGK	0	4	0	10.66
P17844	DDX5	0	4	0	11.89
P11940	PABPC1	5	4	11.16	9.75
Q9NRZ9	HELLS	1	4	1.31	5.01
Q14318	FKBP8	0	4	0	10.44
Q92692	NECTIN2	0	4	0	6.13
P61978	HNRNPK	1	4	3.67	10.15
P26641	EEF1G	0	4	0	9.15
Q06124	PTPN11	0	4	0	6.07
P51114	FXR1	1	4	1.29	5.15
P02545	LMNA	1	4	1.51	7.83
Q96P63	SERPINB12	1	4	2.47	8.64
Q06830	PRDX1	0	4	0	19.1
P05023	ATP1A1	0	4	0	4.3
P15311	EZR	1	4	1.37	5.63
Q8NFF5	FLAD1	0	4	0	5.79
Q14694	USP10	0	4	0	5.26
P22102	GART	0	4	0	3.66
O95801	TTC4	0	4	0	9.82
P40227	CCT6A	0	4	0	6.97
P60842	EIF4A1	0	4	0	13.05
P28482	MAPK1	0	3	0	7.78
P61221	ABCE1	0	3	0	6.51

Q15365	PCBP1	1	3	3.09	14.89
Q8TCT9	HM13	0	3	0	8.75
P23526	AHCY	0	3	0	7.41
O95297	MPZL1	0	3	0	11.9
P12004	PCNA	1	3	4.98	10.73
O95433	AHSA1	1	3	2.66	8.28
Q6YN16	HSDL2	0	3	0	6.94
P07477	PRSS1	1	3	3.24	15.38
Q9BTE3	MCMBP	1	3	1.25	4.67
P54886	ALDH18A1	0	3	0	4.15
P23381	WARS	0	3	0	5.31
Q9BQ70	TCF25	0	3	0	3.85
Q9H6S3	EPS8L2	0	3	0	4.48
P50416	CPT1A	0	3	0	4.4
P26639	TARS	0	3	0	3.73
Q08380	LGALS3BP	4	3	7.18	5.47
Q86YZ3	HRNR	5	3	3.05	1.44
P49327	FASN	3	3	1.31	1.31
Q07666	KHDRBS1	2	3	2.48	4.74
O75695	RP2	1	3	0.43	7.14
P61163	ACTR1A	1	3	3.99	10.9
P05141	SLC25A5	1	3	2.68	9.73
Q4G176	ACSF3	0	3	0	5.56
P30679	GNA15	0	3	0	9.89
P04062	GBA	0	3	0	4.48
P06733	ENO1	0	3	0	9.91
P52597	HNRNPF	1	3	5.54	7.47
Q96PK6	RBM14	1	3	1.79	4.93
P19086	GNAZ	0	3	0	10.99
P08865	RPSA	2	3	7.12	12.88
Q92973	TNPO1	0	3	0	4.01
O60884	DNAJA2	1	3	1.7	6.8
P33992	MCM5	5	3	5.99	4.77
O14929	HAT1	0	3	0	7.16
Q13363	CTBP1	0	3	0	6.36
Q86Y56	DNAAF5	0	3	0	2.92
Q14974	KPNB1	0	3	0	4.11
Q14160	SCRIB	1	3	0.55	2.64
Q9NSD9	FARSB	0	3	0	4.75
Q05086	UBE3A	0	3	0	3.89
P06748	NPM1	2	3	6.12	6.12
O00148	DDX39A	0	3	0	7.49
Q13838	DDX39B	0	3	0	7.48

P05090	APOD	1	3	3.7	14.29
P02786	TFRC	0	3	0	4.87
P11166	SLC2A1	1	2	2.03	5.08
Q96QK1	VPS35	0	2	0	3.02
Q99536	VAT1	0	2	0	2.8
Q13428	TCOF1	0	2	0	1.28
Q6NUK1	SLC25A24	0	2	0	4.4
P50570	DNM2	0	2	0	2.07
P41091	EIF2S3	0	2	0	4.45
Q9Y5A9	YTHDF2	2	2	3.97	3.97
Q86X55	CARM1	1	2	2.14	3.62
P35222	CTNNB1	0	2	0	3.33
P48643	CCT5	0	2	0	3.33
P27361	MAPK3	0	2	0	7.65
Q9Y4W6	AFG3L2	0	2	0	2.76
P63092	GNAS	0	2	0	7.61
Q9NP79	VTA1	1	2	7.82	7.17
P0DOX5	1 SV	1	2	2.67	6.01
Q16186	ADRM1	0	2	0	6.14
Q9Y305	ACOT9	1	2	2.28	4.33
P26599	PTBP1	1	2	1.51	3.58
Q14240	EIF4A2	0	2	0	5.9
O75794	CDC123	0	2	0	7.74
Q2VIR3	EIF2S3B	0	2	0	4.45
P50453	SERPINB9	0	2	0	5.59
O00429	DNM1L	0	2	0	2.58
P30566	ADSL	0	2	0	4.55
P63096	GNAI1	0	2	0	18.08
Q9H5V8	CDCP1	0	2	0	2.63
Q99816	TSG101	0	2	0	5.38
Q12913	PTPRJ	0	2	0	1.57
P30101	PDIA3	8	2	17.43	4.36
Q96EY1	DNAJA3	2	2	5.83	5.83
Q9BQA1	WDR77	8	2	15.79	7.89
P61619	SEC61A1	0	2	0	4.2
Q14498	RBM39	0	2	0	4.72
P08754	GNAI3	0	2	0	14.41
Q9ULV4	CORO1C	0	2	0	3.8
P30519	HMOX2	0	2	0	6.33
P56470	LGALS4	0	2	0	7.12
Q5JWF2	GNAS	0	2	0	2.89
P68371	TUBB4B	2	2	20.9	33.71
Q9NZL9	MAT2B	0	2	0	7.49

A8K2U0	A2ML1	0	2	0	2.2
Q8WVX9	FAR1	0	2	0	4.85
Q2TAA5	ALG11	0	2	0	4.27
P01876	IGHA1	0	2	0	6.8
O75131	CPNE3	0	2	0	4.66
Q9UBM7	DHCR7	0	2	0	3.37
Q9BXW7	HDHD5	0	2	0	6.15
P31944	CASP14	1	2	4.13	7.44
Q9Y6E2	BZW2	0	2	0	4.53
O15427	SLC16A3	0	2	0	4.3
P53992	SEC24C	0	2	0	2.47
P02788	LTF	0	2	0	2.96
Q9UJZ1	STOML2	1	2	4.49	7.3
P39656	DDOST	1	2	1.97	4.39
P01857	IGHG1	1	2	3.64	8.18
Q00325	SLC25A3	1	2	3.31	6.08
Q9UBB4	ATXN10	0	2	0	5.05
Q9UN37	VPS4A	0	2	0	5.26
Q15366	PCBP2	0	2	0	12.05
Q05193	DNM1	0	2	0	2.08
P23921	RRM1	0	2	0	3.66
Q7Z2W4	ZC3HAV1	0	2	0	2.11
P07339	CTSD	2	2	4.37	4.37
Q15293	RCN1	1	2	2.11	6.04
Q13451	FKBP5	0	2	0	4.16
P06702	S100A9	1	2	7.02	18.42
P08243	ASNS	0	2	0	4.46
Q13557	CAMK2D	2	2	5.01	5.01
P17812	CTPS1	0	2	0	3.05
P29992	GNA11	0	1	0	9.47
